# Social exclusion and psychopathology in LGBTQ+ communities: a neuropsychosocial review

**DOI:** 10.3389/fsoc.2025.1638766

**Published:** 2025-07-24

**Authors:** Stergios Kaprinis, Anastasios Charalampakis

**Affiliations:** Second Department of Psychiatry, Faculty of Health Sciences, School of Medicine, Aristotle University of Thessaloniki, Thessaloniki, Greece

**Keywords:** minority stress, internalized stigma, rejection sensitivity, emotional dysregulation, intersectionality, neurobiology

## Abstract

LGBTQ+ individuals are disproportionately affected by depression, anxiety, and suicidal ideation, primarily due to persistent social exclusion, prejudice, and systemic discrimination rather than any inherent psychopathology. This review synthesizes contemporary theoretical frameworks, including the Minority Stress Model, the Psychological Mediation Framework, and the Rejection Sensitivity Model, to examine the internalization of systemic marginalization and its manifestation as psychological distress. Significant mediators, such as internalized stigma, emotional dysregulation, and rejection sensitivity, are investigated alongside structural determinants, such as familial rejection and intersectional oppression. Contemporary insights from social psychology, psychiatry, and neuroscience were used in this study. Neurobiological data indicate that chronic minority stress modifies the limbic–prefrontal circuitry, disrupts the hypothalamic– pituitary–adrenal (HPA) axis, and increases allostatic load. The implications for clinical practice and public health were analyzed, emphasizing the importance of community-based resilience initiatives, inclusive policy reforms, and LGBTQ+-affirmative therapy. The article concludes by outlining the theoretical constraints and proposing future avenues for participatory and multidisciplinary studies.

## Introduction

LGBTQ+ individuals experience disproportionately high rates of mental health disorders, including significantly elevated incidences of depression, anxiety, substance abuse, and suicidality, compared to heterosexual and cisgender populations (Plöderl and Tremblay, 2015; Semlyen et al., 2016; Valentine and Shipherd, 2023). These disparities are primarily attributable to prolonged exposure to psychosocial stressors such as systemic discrimination, interpersonal rejection, identity invalidation, and limited access to supportive resources, rather than intrinsic psychological susceptibility (Meyer, 2003; Hatzenbuehler, 2009; Pachankis and Bränström, 2018). In contemporary psychiatric and psychological research, LGBTQ+ mental health is increasingly conceptualized as a syndemic phenomenon, where various structural and social stressors converge to exacerbate psychological distress rather than being viewed solely as an individual issue (Stall et al., 2003; Logie et al., 2020). The Minority Stress Model is a critical framework that posits that distal stressors (e.g., violence and discrimination) and proximal stressors (e.g., internalized stigma and concealment) related to marginalized identity substantially affect the mental health outcomes of LGBTQ+ individuals (Frost and Fingerhut, 2021; Meyer, 2003). These chronic pressures often persist throughout an individual's life and exceed the expectations of non-minorities. Recent meta-analyses corroborated these findings. For example, transgender individuals exhibit alarmingly high rates of clinical depression (up to 50%), suicidal ideation (50–70%), and suicide attempts (40% or more) (Bauer et al., 2021; Toomey et al., 2022), and LGB adults are twice as likely to experience mental health disorders (King et al., 2008). These findings are consistent across many geographical and cultural contexts, suggesting that the underlying mechanisms are based on shared experiences of social marginalization and minority stress (White Hughto and Reisner, 2016). Furthermore, social psychology emphasizes that social comparison, rejection sensitivity, and sense of belonging are significant mediators between exclusionary contexts and mental illness in LGBTQ+ communities (Downey and Feldman, 1996; Pachankis et al., 2021). The structural impact of anti-LGBTQ+ legislation, heteronormative policies, and inadequate healthcare training exacerbates these issues, thereby perpetuating institutionalized stigma (Flores et al., 2020). An integrative multidisciplinary approach is necessary to understand these complex pathways. Synthesizing insights from clinical psychiatry, social psychology, and critical structural theories can provide a comprehensive analysis of the impact of societal influence on the resilience and mental health of LGBTQ+ individuals. This approach is essential for creating culturally attuned interventions, inclusive public health policies, and supportive mental health services, as well as for advancing scientific knowledge.

## Conceptual framework

### Minority Stress Theory

**Figure 1:**
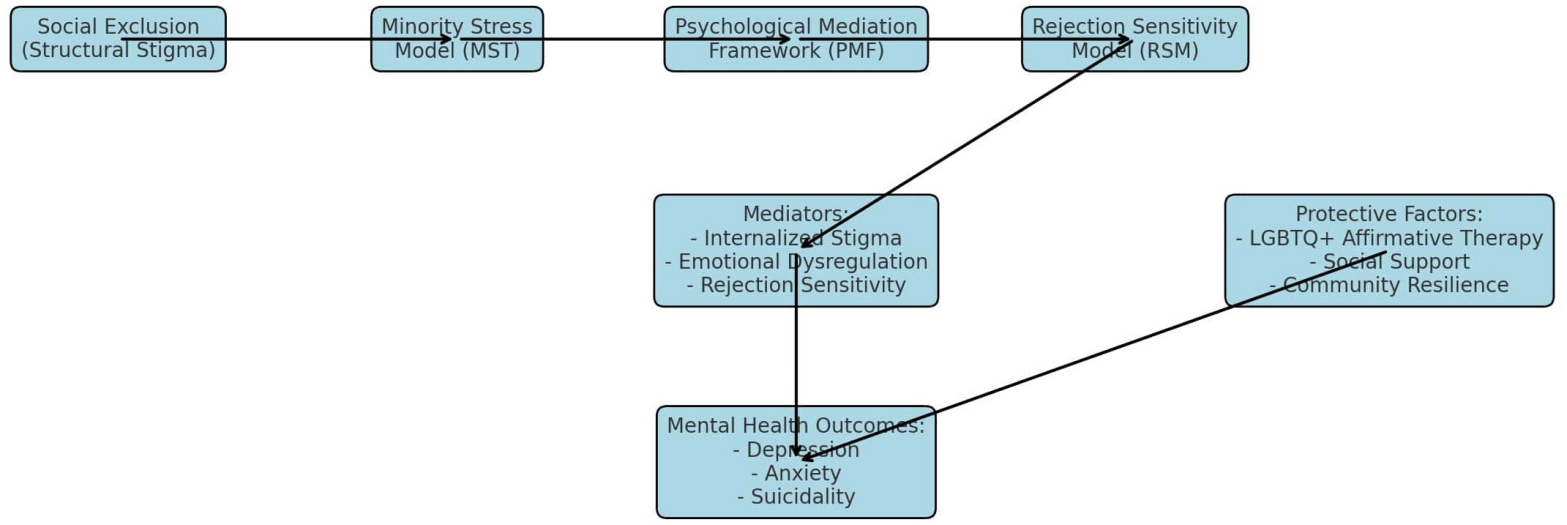
Conceptual integration of Minority Stress Theory (MST), Psychological Mediation Framework (PMF), and Rejection Sensitivity Model (RSM).

The Minority Stress Theory (MST) was developed by Ilan. Meyer (2003) served as a pivotal theoretical framework for elucidating the mental health disparities observed among LGBTQ+ individuals. This theory asserts that sexual and gender minorities face persistent societal pressures such as institutional discrimination, structural inequality, and enduring stigma, rather than pressures stemming from their identities. These stressors are directly associated with increased psychiatric morbidity, including depression, anxiety, and suicidality, and extend beyond ordinary stress (see Figure 1).

The paradigm posits that minority stress is multidimensional and consists of two distinct categories: distal stresses, which include overt manifestations of violence, harassment, and discrimination, such as hate crimes and identity-based termination of employment.

Proximal stressors encompass the imperative to conceal one's identity in hazardous situations, expectations of rejection, heightened vigilance, and internalized stigma, including internalized homophobia and transphobia.

These stressors collectively contribute to a substantial allostatic load, which signifies cumulative “wear and tear” on the body resulting from prolonged stress exposure. This burden has been empirically associated with heightened systemic inflammation, disruption of the hypothalamic– pituitary– adrenal (HPA) axis, and altered cortisol levels Doyle and Pachankis, 2016; Doyle and Molix, 2021).

The concept has been validated across various contexts and identities through subsequent empirical extensions of MST. Internalized stigma and rejection sensitivity independently predicted major depressive disorder and anxiety in LGBTQ+ individuals, as demonstrated by a systematic review conducted by Sattler et al. (2017). For adolescents, familial rejection and concealment constitute significant proximal stressors, correlating with a substantially increased likelihood of suicidal ideation (Toomey et al., 2019; Russell and Fish, 2020).

Moreover, the minority stress theory (MST) is supported by neuroscientific evidence demonstrating that social exclusion and stigma activate the same neural pathways as physical pain, specifically the anterior insula and dorsal anterior cingulate cortex (Eisenberger, 2015). This convergence underscores the embodied nature of minority stress and its potential to induce psychological and physiological dysregulations.

MST fundamentally encompasses both the structural and intrapersonal aspects of marginalization. Structural stigma, exemplified by discriminatory legislation, heteronormative educational systems, and unequal access to healthcare, creates environments that marginalize, survey, or eliminate LGBTQ+ individuals, thereby intensifying minority stress (Hatzenbuehler, 2016). Nevertheless, despite persistent levels of interpersonal stigma, LGBTQ+ individuals report significantly improved mental health outcomes in countries or regions characterized by diminished structural stigma (Pachankis et al., 2021).

Despite its widespread application, Minority Stress Theory (MST) has evolved to address intersectionality and context-specific variations.

Researchers emphasize the interplay of sexual and gender identity with race, ethnicity, socioeconomic status, immigration history, disability status, and other variables, resulting in intricate manifestations of compound minority stress (Balsam et al., 2011; Bowleg, 2020). These advancements suggest that MST should be employed along with a comprehensive biopsychosocial model that acknowledges the interconnected nature of resilience and injustice.

There is considerable evidence indicating the protective function of affirmative environments such as LGBTQ+ community networks, inclusive workplaces, and supportive educational institutions. Affirmation of one's identity, cultivation of social connections, and receipt of validation within these contexts can improve mental health outcomes and alleviate the adverse effects of minority stress (Snapp et al., 2015; Bauer et al., 2021).

### Framework for psychological mediation

Hatzenbuehler (2009) introduced the Psychological Mediation Framework (PMF) to address the limitations inherent in the original Minority Stress Theory, which predominantly concentrated on identity-related and external stressors. This framework represents a substantial advancement in clinical and intervention science, as it conceptualizes the relationship between structural stigma and mental health as being mediated by modifiable psychological processes rather than as a direct, linear association.

The PMF posits that exposure to structural stigma, characterized by institutional policies, cultural norms, and societal conditions that limit the opportunities, resources, and wellbeing of marginalized groups, adversely affects mental health over time through internal psychological processes. Hatzenbuehler identified three primary mediators.

Emotional dysregulation:

1) Maladaptive cognitive processes, including rumination and negative attributional styles
2) Social and interpersonal challenges, especially feelings of rejection and isolation.

#### Dysregulation of emotions

Individuals who identify as LGBTQ+ and experience stigma, rejection, or need for concealment often encounter difficulties in regulating their emotions, particularly in social or high-pressure contexts. Such challenges are characterized by heightened reactivity, repression, and difficulty in restoring emotional equilibrium following distress, which is indicative of emotional dysregulation. Research indicates that the association between discrimination and the symptoms of anxiety and depression is mediated by emotional dysregulation (Feinstein et al., 2012; Hatzenbuehler and Pachankis, 2016). Furthermore, increased emotional dysregulation has been directly linked to an increase in suicidality among transgender youth (Zalewski et al., 2021).

#### Maladaptive cognitive assessment

This involves holding negative views of oneself, others, and future events, which are often shaped by prolonged marginalization or invalidation.

Gender and sexual minorities may utilize ruminative coping mechanisms, accept adverse stereotypes, or adopt pessimistic explanatory frameworks. Empirical research indicates that LGBTQ+ individuals experiencing heightened structural stigma are more prone to negative self-schemas, subsequently elevating their risk of major depressive disorder and diminishing self-esteem (Newcomb and Mustanski, 2010; Pachankis et al., 2015).

Recent advancements in affective neuroscience substantiate these findings, demonstrating that extended exposure to stigma alters neural responses to social feedback. This results in heightened amygdala activation and diminished prefrontal regulation, which serve as identifiable markers of sustained emotional hyperactivity and distorted threat perception (Burghy et al., 2012; Cloitre et al., 2022).

#### Social exclusion and rejection sensitivity

The deterioration of social connections is another critical concern. LGBTQ+ individuals frequently face peer rejection, diminished familial support, and estrangement due to concealment, all of which erode protective social bonds. This erosion may result in chronic social isolation, which is recognized as a precursor to depression, substance abuse, and suicidal ideation (Ryan et al., 2010; Bauer et al., 2021). The interplay between psychopathology and discrimination is mediated by rejection sensitivity, characterized by increased anticipation of and sensitivity to rejection, serving as a cognitive-affective vulnerability factor (Pachankis et al., 2020; Feinstein, 2019).

Longitudinal studies have demonstrated that these mediators are both adaptable and responsive to intervention. Psychotherapeutic approaches that focus on emotion regulation (e.g., Dialectical Behavior Therapy), cognitive restructuring (e.g., Cognitive Behavioral Therapy), and interpersonal connections (e.g., group-based affirming therapies) have been shown to significantly alleviate distress among LGBTQ+ individuals, particularly when implemented in identity-affirming settings (Craig et al., 2021; Flentje et al., 2020).

The PMF model elucidates the profound penetration of stigma, transforming external adversity into internal suffering rather than merely documenting discrepancies. It establishes a connection between structural criticism and clinical action by focusing on modifiable mediators, thereby facilitating the development of therapies that mitigate the psychological effects of marginalization.

### Framework of rejection sensitivity

The Rejection Sensitivity Model (RSM), formulated by Downey and Feldman in 1996, delineates a cognitive-affective processing tendency wherein individuals anticipate, perceive, and respond intensely to rejection.

Originally conceptualized for romantic relationships and attachment, this paradigm was adapted to address marginalized groups that experience persistent rejection due to their identity, particularly LGBTQ+ individuals.

#### Essential RSM assumptions among LGBTQ+ communities

LGBTQ+ individuals may develop heightened vigilance toward rejection signals, even in ambiguous contexts, as a result of repeated experiences of stigmatization, bullying, misgendering, familial rejection, or social invalidation (Pachankis et al., 2015; Feinstein, 2019). This “expectancy bias” intensifies the perception of social threat, thereby increasing individuals' vulnerability to:

- Anxiety disorders, specifically social anxiety.
- Indicators of depression.
- Social avoidance and retreat.
- Suppressing one's voice and concealing one's identity.

Internalizing fear of rejection can result in maladaptive coping strategies, such as emotional repression, avoidance of personal connections, or pre- emptive withdrawal from social and professional contexts.

#### Empirical evidence

Research has increasingly supported the predictive validity of RSM in sexual and gender minority populations.

Feinstein et al. (2012) demonstrated that gay and bisexual men exhibiting elevated levels of rejection sensitivity were significantly more prone to report symptoms of anxiety and sadness, irrespective of their actual experiences of discrimination. Pachankis et al. (2021) demonstrate that the association between minority stress—encompassing identity concealment and structural stigma—and psychological distress among men identifying as sexual minorities is mediated by rejection sensitivity.

Rejection sensitivity is correlated with an elevated risk of suicidal ideation among transgender individuals, particularly when compounded by familial rejection and gender-based invalidation (Rodríguez Seijas et al., 2021).

Hatzenbuehler et al. (2015) contend that rejection sensitivity may intensify the impact of internalized homophobia by causing affective dysregulation and promoting a negative self-concept.

#### Correlates in neurocognitive function

Recent neuroscientific research substantiates the theoretical assertions of the model by demonstrating a connection between rejection sensitivity and increased activity in the amygdala and anterior cingulate cortex, regions implicated in social pain and threat perception (Eisenberger and Lieberman, 2004). Burklund et al. (2007) further contended that individuals with heightened rejection sensitivity exhibit diminished prefrontal regulatory control, indicating a reduced ability to cognitively reframe experiences of rejection.

#### RSM as a mechanism of minority stress

Rejection sensitivity functions as a proximal mediator within the Minority Stress Model (Hatzenbuehler, 2009; Meyer, 2003), a psychological mechanism that internalizes external stressors including discrimination and legal inequality. This results in psychopathology, interpersonal strain, and dysregulation of affect. The relationship between rejection sensitivity and structural stigma can be conceptualized as a feedback loop: prolonged exposure heightens sensitivity, adversely affects mental health, and diminishes protective interactions with others.

#### Consequences for clinical and public health

Rejection sensitivity is the primary focus of intervention because of its modifiability.

The primary objective of affirmative cognitive behavioral therapy (CBT) is to restructure threat assessments and enhance social self-efficacy. These therapeutic approaches have shown promising outcomes in mitigating anxiety and depression associated with rejection (Flentje et al., 2015, 2020).

Group-based therapies that foster community connection and peer validation appear to alleviate the consequences of rejection sensitivity, particularly among LGBTQ+ youths (Craig et al., 2021).

## Mental health consequences and social marginalization

A significant determinant of negative mental health outcomes within LGBTQ+ populations is social exclusion, which is characterized by the systematic denial of full participation in the societal, legal, and cultural domains. Unlike temporary interpersonal stress, minority stress emerges from systemic and institutional exclusion such as legal discrimination, exclusion from healthcare protocols, and educational marginalization. This form of stress undermines emotional resilience, damages one's self-concept, and increases susceptibility to psychiatric morbidity (White Hughto and Reisner, 2016; Hatzenbuehler, 2015).

### The economic depression

Epidemiological research has consistently demonstrated that the prevalence of depressive symptoms, major depressive episodes, and clinical depression is markedly higher among LGBTQ+ individuals than among their cisgender heterosexual counterparts. Meta-analyses have indicated that depression is 2.5–3 times more common among LGB individuals (King et al., 2008; Plöderl and Tremblay, 2015), with transgender individuals exhibiting even higher rates. Prevalence estimates vary according to age and geographical location (Bauer et al., 2021; Valentine and Shipherd, 2023).

The significant routes comprise the following:

Social instability, financial strain, and a sense of reduced belonging stemming from systemic exclusion from marriage, adoption, and employment rights.

Ongoing concealment or invalidation of one's identity is associated with internalized shame, despair, and negative self-perception (Newcomb and Mustanski, 2010).

Ryan et al. (2010) asserted that familial and communal rejection, particularly during adolescence, is a significant predictor of early onset depression and concurrent substance use.

Neuroimaging findings in depressed LGBTQ+ individuals suggest that persistent social stress can alter the activity of the hypothalamic–pituitary– adrenal (HPA) axis and reduce hippocampal volume (Kirst et al., 2021).

### Anxiety disorders

A range of anxiety disorders are closely linked to extended exposure to adverse circumstances, such as misgendering, insecurity in public spaces, and microaggressions stemming from homophobia or transphobia. These conditions include the following.

Generalized Anxiety Disorder (GAD)
Social Anxiety Disorder
Anxiety Disorder
Fear of agoraphobia

These symptoms frequently emerge during early adolescence, a developmental stage marked by identity formation and increased vulnerability to peer evaluation. Empirical evidence suggests that experiences of misgendering and the use of incorrect pronouns lead to heightened physiological arousal and autonomic dysregulation in transgender and non-binary youths, thereby elevating their risk of developing chronic anxiety disorders (McLemore, 2018; Restar, 2022).

Furthermore, anticipatory anxiety induced by environmental insecurity in settings such as workplaces, educational institutions, and medical facilities can result in the following.

- Excessive vigilance
- Actions of avoidance
- an increase in maladaptive coping strategies, including substance abuse and detachment.

Persistent anticipatory anxiety and avoidance can impede social, intellectual, and professional functioning as defined by the DSM-5-TR (American Psychiatric Association, 2022).

### Suicide

The significantly elevated risk of suicidal ideation, planning, and attempts constitutes the most alarming mental health disparity in the LGBTQ+ community. Research indicates that LGB youth are 3–5 times more likely to contemplate or attempt suicide than their heterosexual peers (Marshal et al., 2011; Russell and Fish, 2020). The lifetime suicide attempt rate among transgender individuals is ~40–45%, a statistic unmatched in other clinical populations (Haas et al., 2014; The Trevor Project, 2022).

The contributing factors include the following.

- Prolonged and early ostracism by friends, family, or religious organizations.
- Exposure to hate speech and anti-LGBTQ+ legislation.
- invalidation of identity; and
- internalized stigma.

Longitudinal studies have shown that inadequate provision of gender- affirming care increases the risk of suicide, whereas its provision reduces this risk by up to 70% (Turban et al., 2020).

Within the suicidogenic process, social exclusion serves both as a direct catalyst and contextual element. According to the interpersonal psychological theory of suicide (Joiner, 2005), the likelihood of suicidal behavior is markedly heightened by perceived burdensomeness and thwarted belongingness, factors frequently encountered by marginalized LGBTQ+ individuals.

## Interpersonal and structural mediators

Societal exclusion serves as a broader context for mental health disparities among LGBTQ+ individuals; however, interpersonal and intrapsychic mechanisms influence the pathway from stigma to psychopathology. These mediators act as critical intervention points explaining why individuals subjected to similar structural conditions experience varying outcomes. This section delineates three principal areas: intersectionality, social support, and internalized stigma.

### Repression of identity and internalized stigmatization

The integration of negative societal perceptions regarding non-normative sexual orientations and gender identities into an individual's self-concept is termed internalized stigma, which is also referred to as internalized homophobia or transphobia. When LGBTQ+ individuals are exposed to hostile environments, they may begin to diminish their own identities, potentially resulting in the following:

- Persistent humiliation
- Inadequate self-worth and self-reproach
- declining to articulate one's identity repression of emotions

Suicidality, anxiety, and depression are closely connected to this process (Newcomb and Mustanski, 2010; Feinstein, 2020). A longitudinal study conducted by Meyer et al. (2013) demonstrated that heightened levels of internalized stigma correlated with poorer mental health outcomes, even when controlling for external discrimination. The act of concealing one's identity, a suppression strategy employed to evade discrimination, exacerbates social withdrawal and cortisol dysregulation, both of which contribute to increased mental vulnerability (Doyle et al., 2015; Doyle and Molix, 2021).

Neurobiological evidence indicates that internalized stigma exacerbates self-blame and emotional avoidance by modifying the brain regions associated with emotion regulation and self-referential processing, such as the medial prefrontal cortex (Kirst et al., 2021).

### The safeguarding function of social support

Social support, particularly from peers, families, and affirming groups, is among the most well-documented protective factors in research on LGBTQ and mental health. Social support served as a protective mechanism.

Enhancing emotional regulation:

- alleviating feelings of isolation and burden
- fostering the development of a constructive identity
- Enhancing accessibility of healthcare and support resources.

Ryan et al. (2010) identified that LGBTQ+ youth who experienced familial acceptance were three times more likely to engage in substance use, six times more likely to suffer from severe depression, and eight times more likely to attempt suicide than those who encountered familial rejection.

Similarly, peer support from online communities or Gender and Sexuality Alliances (GSAs) has been correlated with diminished internalized stigma, enhanced resilience, and increased participation in school activities (Craig et al., 2021; The Trevor Project, 2022).

Familial rejection is a significant risk factor. Adolescents who endure maltreatment or rejection from their guardians due to their identity are more predisposed to:

- homelessness; substance dependence; PTSD symptoms; chronic suicidality

Chronic suicidality (Keuroghlian et al., 2017; Snapp et al., 2015).

The mitigation of structural stigma can be achieved by promoting social connections. In states with rigorous anti-transgender legislation, transgender individuals who perceive significant community support report lower depression levels (Bauer et al., 2021).

### Compounded exclusion and intersectionality

First introduced by Crenshaw (1991), the concept of ‘intersectionality' elucidates the interconnected and cumulative effects of various social stratification characteristics, such as race, gender, sexual orientation, class, and disability. The mental health disparities experienced by LGBTQ+ individuals are exacerbated for those who also belong to racially, ethnically, or socioeconomically marginalized communities due to the following reasons:

- Multifaceted discrimination (e.g., racism and homophobia).
- Systemic poverty (e.g. insufficient educational financing or precarious housing).
- Invisibility or cultural stigma within LGBTQ+ and ethnic communities.

A 2022 national study conducted by the Human Rights Campaign indicated that Black LGBTQ+ children are subjected to elevated levels of neighborhood violence, food insecurity, and police harassment, all of which are significantly associated with post-traumatic stress disorder (PTSD) and suicidal ideation. Furthermore, Velez et al. (2020) identified that transgender Latinx individuals encounter discrimination within healthcare settings and face restricted access to affirming services, leading to a heightened prevalence of mental health issues.

Intersectional oppression intensifies cumulative disadvantages by operating vertically, across various systems, and horizontally across different identities (Bowleg, 2020). The absence of an intersectional perspective in research and interventions may obscure the complex realities faced by the most vulnerable subgroups within the LGBTQ+ communities.

## Neurobiological pathways

Extended exposure to societal stressors and stigma, as encountered by LGBTQ+ individuals, exerts quantifiable effects on both the physiological and neurological systems. It alters immunological, endocrine, and neurological pathways, thereby increasing the susceptibility to mood disorders, anxiety, and trauma-related conditions. Recent advancements in neuroimaging and psychoneuroendocrinology have offered compelling evidence that social exclusion is biologically embedded.

### Dysregulation of the hypothalamic–pituitary–adrenal axis

The hypothalamic–pituitary–adrenal (HPA) axis constitutes the central neuroendocrine system responsible for modulating the body's response to stress. Sustained activation of the HPA axis, precipitated by persistent social evaluative threats such as rejection, discrimination, or concealment, results in the following:

- increased concentrations of basal cortisol
- reduced cortisol variability across the day
- Reduced reactivity to abrupt stressors.

These modifications have been consistently documented in LGBTQ+ individuals experiencing minority stress (Doyle and Molix, 2021; Hatzenbuehler and McLaughlin, 2014). Dysregulated cortisol secretion has been associated with impaired emotional regulation, cognitive rigidity, and somatic comorbidities including immune suppression and metabolic syndrome.

### Decrease in hippocampal volume

Chronic stress has been shown to decrease the volume of the hippocampus, a critical brain region involved in evaluating contextual threats, consolidating memories, and regulating the feedback of the HPA axis. This phenomenon has been substantiated by neuroimaging studies (McEwen, 2000; Sapolsky, 2004). A reduction in hippocampal volume has been linked to

Major depressive disorder (MDD).
Post-traumatic stress disorder (PTSD).

#### Challenges faced in early life by LGBTQ+ individuals

A recent meta-analysis by Kirst et al. (2021) revealed significant hippocampal shrinkage in individuals subjected to chronic minority stress. This effect was particularly pronounced among transgender youths who had experienced victimization at school or familial rejection.

#### Disruption of amygdala-prefrontal connectivity

The prefrontal cortex (PFC) is responsible for emotion regulation and cognitive control, whereas the amygdala plays a crucial role in identifying emotional significance and assessing potential threats. In a healthy system, the prefrontal cortex inhibits heightened responses of the amygdala.

However, when exposure to social threats becomes chronic, this regulatory circuit may become dysregulated, leading to the following:

- increased amygdala activation in response to social stimuli

Hypoconnectivity of the amygdala and medial prefrontal cortex.

- diminished capacity to generalize dangers; and
- eradication of fear.

These patterns have been observed among individuals who are gender- diverse and experience systematic invalidation, as well as among sexual minorities who encounter significant perceived rejection (Burklund et al., 2007; Jovanovic et al., 2020). Consistent with models of rejection sensitivity and hypervigilance, functional MRI studies have indicated that LGBTQ+ individuals demonstrate increased brain sensitivity to ambiguous social cues (Eisenberger and Lieberman, 2004).

### Systemic effects and allostatic load

Multisystem dysregulation arises from the cumulative impact of chronic stress, a phenomenon known as allostatic load. This includes:

- Inflammation (elevated TNF-α and IL-6 levels).
- mitochondrial impairment and oxidative stress imbalance in the autonomic nervous system (elevated sympathetic tone).

The biological mechanisms in question can result in cardiovascular disease, diabetes, and premature aging while also elevating the risk of mental illness among LGBTQ+ individuals (Doyle et al., 2015; Doyle and Molix, 2021).

The enduring biological effects of structural exclusion are demonstrated by the reduction in telomere length and acceleration of epigenetic aging in young individuals experiencing minority stress and socioeconomic adversity (Colich et al., 2020).

## Consequences for clinical and public health

The growing body of evidence linking minority stress, social exclusion, and psychopathology within LGBTQ+ populations underscores the need for a comprehensive public health and therapeutic strategy that addresses both individual distress and systemic factors that influence mental health. For such an intervention to be effective, it must be at the clinical, institutional, community, and policy levels.

### Affirmative therapies for LGBTQ+ individuals

Affirmative psychotherapy is recognized as the best practice in mental healthcare, as it acknowledges and validates LGBTQ+ identities (American Psychological Association, 2021). Instead of pathologizing identity, these therapeutic approaches consider the sociopolitical context of LGBTQ+ distress and emphasize the following:

- Deconstructing internalized stigma
- enhancing the integration of identities
- Fostering resilience in the face of rejection

Fostering connections within communities.

Randomized controlled trials have demonstrated the substantial efficacy of emotion-focused therapies and LGBTQ+ affirmative cognitive behavioral therapy (CBT) in mitigating the symptoms of PTSD, anxiety, and depression among sexual and gender minorities (Flentje et al., 2015, 2020). These interventions significantly enhance treatment adherence, relationship quality, and self-esteem, particularly among transgender individuals (Craig et al., 2021).

The association between access to physical and mental healthcare is underscored by the relationship between gender-affirming medical interventions, such as hormone therapy and surgical procedures, and the reduction in suicidality alongside improvements in psychological wellbeing (Turban et al., 2020).

### Structural reforms and policy interventions

Personalized therapy alone is insufficient to address the harm inflicted by institutional exclusion, adversarial public policies, or legal discrimination. Therefore, structural reforms of this nature should be incorporated into public health strategies.

Legislation safeguards gender identity and sexual orientation against discrimination in the domains of employment, housing, healthcare, and education.

- Comprehensive data collection to accurately portray LGBTQ+ identities in clinical documentation and epidemiological research.

Funding for mental health services specifically designed for the LGBTQ+ population, particularly in disadvantaged regions.

Mandatory cultural competency training for public servants, educators, and healthcare professionals.

Research indicates that mental health disparities within LGBTQ+ communities are consistently less pronounced in countries and regions that implement inclusive policies, such as legal gender recognition, same-sex marriage, and protection against conversion therapy, compared with those with regressive legislation (Russell and Fish, 2016, 2020).

### Empowerment and resilience within the community

Community-based initiatives in conjunction with therapeutic interventions and legislative measures are crucial for mitigating the impact of social isolation. These initiatives facilitate the following.

- Social engagement via peer support groups, youth drop-in facilities, and LGBTQ+ organizations.

Engagement in civic activities and the cultivation of leadership skills to enhance political agency and self-efficacy.

- Culturally appropriate coping methods, particularly for intersectionally oppressed groups such as immigrants, people of color, disabled people, and LGBTQ+ individuals.

Research indicates that interventions such as Q Chat Space, the Trevor Project's Lifeline, and the Black Trans Advocacy Network have beneficial effects on identity pride, suicide prevention, and emotional wellbeing, particularly among young individuals (Craig et al., 2021; The Trevor Project, 2022).

By reconceptualizing LGBTQ+ mental health as a political and human rights issue, as well as a treatment concern, these programmes foster individual recovery and collective resilience.

## Critical evaluation and future outlook

This review elucidates the intricate mechanisms that connect social exclusion to mental health disparities within LGBTQ+ populations by synthesizing three principal theoretical frameworks: the Rejection Sensitivity Model (RSM), Minority Stress Model (MSM), and Psychological Mediation Framework (PMF). Although these models have significantly advanced our comprehension of identity-related stress and psychopathology, a comprehensive analysis has revealed conceptual limitations and potential areas for refinement.

### Theoretical benefits and limitations

The Minority Stress Model serves as a foundational framework for understanding the effects of both proximal and distal stressors on the mental health of LGBTQ+ individuals. Nevertheless, it frequently underemphasizes the significance of protective factors, such as identity pride, community affiliation, and adaptive coping strategies, thereby prioritizing pathophysiology over resilience. Additionally, the model is rooted in an individualistic, predominantly Western paradigm that may not be applicable to collectivist or non-Western contexts.

The Psychological Mediation Framework enhances the Minority Stress Model by identifying cognitive-affective mediators such as emotion dysregulation and social isolation. Nevertheless, it lacks integration with sociocultural and developmental trajectories, including identity development across an individual's lifespan, and the impact of factors such as race, religion, and immigration status on psychological processing.

While the Rejection Sensitivity Model effectively elucidates the emotional ramifications of perceived interpersonal threats, it has not been substantiated through longitudinal research within LGBTQ+ populations, and may underestimate individuals' capacity to modify their sensitivity through community-based or therapeutic interventions.

### Article limitations and recommendations

This review addresses social, neurological, and psychological dimensions; however, it is not comprehensive. Several gaps persist:

- Inadequate emphasis is placed on resilience mechanisms such as queer joy, collective healing, and post-traumatic growth.

There is insufficient representation of asexual, intersex, and non-binary populations whose experiences are not well captured by current frameworks.

Research in the Global South is characterized by distinct structural conditions and identity conceptions and requires global perspectives.

Most of the referenced evidence comes from cross-sectional studies, highlighting the need for longitudinal and experimental designs to establish causation.

### Prospects for continued research

To advance the discipline and improve its clinical and policy significance, future research should undertake the following:

- Integrate intersectionality as a formal construct within stress models to elucidate how LGBTQ+ identities are affected by variables such as race, class, ability, and religion.

Develop ecological frameworks for multilevel analyses that integrate variables at the individual, relational, institutional, and policy levels.

Examine the aggregate impact of minority stress and the potential for post- intervention neurological healing via extensive neurobiological research.

- Analyze defense mechanisms that alleviate the impact of stigma and promote flourishing, including pride, advocacy, and spiritual integration.

Prioritize community-based participatory research (CBPR) to elevate the needs and voices of LGBTQ+ individuals in the design, interpretation, and dissemination of research.

Investigate digital environments, including social media and online peer support, as novel platforms for stress exposure and resilience development.

## Conclusions

The models under evaluation have had a profound influence on contemporary research on LGBTQ and mental health. It is imperative that theoretical frameworks evolve to integrate the concepts of resilience, intersectionality, cultural context, and structural power, thereby remaining responsive to the diversity and complexity inherent in LGBTQ+ lived experiences. To establish systems that promote mental health equity and liberation, it is essential to employ multidisciplinary, justice-oriented, and community-engaged strategies that extend beyond mere documentation of disparities.
